# Innovative methods for using expert panels in identifying implementation strategies and obtaining recommendations for their use

**DOI:** 10.1186/1748-5908-10-S1-A44

**Published:** 2015-08-20

**Authors:** Thomas J Waltz, Byron J Powell, Monica M Matthieu, Matthew J Chinman, Jeffrey L Smith, Enola K Proctor, Laura J Damschroder, JoAnn E Kirchner

**Affiliations:** 1 Department of Psychology, Eastern Michigan University, Ypsilanti, MI 48197, USA; 2 Center for Clinical Management Research and Diabetes QUERI, VA Ann Arbor Healthcare System, Ann Arbor, MI 48105, USA; 3 Center for Mental Health Policy and Services Research Department of Psychiatry, Perelman School of Medicine University of Pennsylvania, Philadelphia, PA 19104, USA; 4 Central Arkansas Veterans Healthcare System, HSR&D and Mental Health Quality Enhancement Research Initiative (QUERI), Little Rock, AR 72114, USA; 5 School of Social Work, College for Public Health & Social Justice, Saint Louis University, St. Louis, MO 63103, USA; 6 VISN 4 MIRECC, VA Pittsburgh Healthcare System, and RAND Corporation, Pittsburgh, PA 15213, USA; 7 Brown School, Washington University in St. Louis, St. Louis, MO 63105, USA; 8 Department of Psychiatry, College of Medicine, University of Arkansas for Medical Sciences, Little Rock, AR 72205, USA

## Introduction

A variety of research questions can be addressed using expert panels to synthesize existing knowledge and issue recommendations. This panel's presentations describe the use of innovative methods for engaging expert panels comprised of implementation scientists and clinical managers in complex recommendation processes to match implementation strategies with evidence based practices in real world service settings as part of the Veterans Health Administration (VA) funded 'Expert Recommendations for Implementing Change' (ERIC) project (QLP 55-025).

## Methods

Powell describes the use of a web-based modified-Delphi processes to obtain expert consensus on a compilation of discrete implementation strategies. Waltz describes the use of a concept mapping method to characterize the interrelationships among the strategies in the compilation. Finally, Matthieu describes the methods used to engage multiple stakeholders to develop a structured recommendation process that applies the implementation strategies to specific practice changes in the VA.

## Results

The innovative sequence of methods used highlights the value of structured tasks that support transparent, quantitative characterizations of expert panel recommendations. The majority of this expert panel's activities involved asynchronous use of a variety of software platforms, reducing logistical barriers often involved in engaging a large panel of experts. Activities that required synchronous consensus meetings also utilized technology to host structured discussions and post-discussion voting that provided participants with real time feedback on the recommendation outcomes.

## Discussion

The sequence of methods employed in the ERIC project can serve as a model for developing context-sensitive expert recommendations for other dissemination and implementation initiatives.

Building expert consensus for characterizing discrete implementation strategies

Efforts to identify, develop, and test implementation strategies have been complicated by the use of inconsistent language and inadequate descriptions of strategies in the scholarly literature. A literature-based compilation of strategies was developed to address this problem (Powell et al., 2012); however, its development was not informed by the participation of a wide-range of implementation and clinical experts. This presentation describes our effort to further refine that compilation for use in the VA by establishing expert consensus on strategy terms, definitions, and categories that can be used to guide implementation research. Purposive sampling was used to recruit an expert panel comprised of implementation science experts and VA clinical managers. Specifically, a reputation-based snowball sampling approach was used in which an initial list of experts was developed by members of the study team. This list included the editorial board of the journal *Implementation Science*, Implementation Research Coordinators from the VA QUERI program, and faculty from the NIH-funded Implementation Research Institute. The Expert Panel was engaged in a three-round modified Delphi process to generate consensus on strategies and definitions. The first and second rounds involved web-based surveys that prompted comments on implementation strategy terms and definitions. The initial survey was seeded with strategy terms and definitions from the Powell et al. (2012) compilation. After each round, iterative refinements were made to the compilation based upon participants' feedback. The third round involved a live, web-based polling process and consensus process that yielded a final compilation of 73 strategies and definitions. This presentation highlights the advantages and challenges associated with using asynchronous and live web-based methods for obtaining wide participation of experts.

Concept mapping: harnessing the power of an expert panel to conceptualize relationships among 73 implementation strategies

After obtaining the compilation of discrete implementation strategies in the earlier phase of the Expert Recommendations for Implementing Change (ERIC) project, we were faced with a practical challenge of how to realistically ask experts to consider 73 different implementation strategies when making recommendations. One strategy to reduce the cognitive burden of a complex multicomponent recommendation development process is to organize strategies by similarity. Concept mapping is a method that allows you to engage an expert panel in a structured task that can be completed asynchronously and online. For this study, expert panel members were given a deck of virtual "cards", each with one of ERIC's 73 implementation strategies. Participants then sorted these cards into piles on the basis of similarity and then rated each strategy in terms of its relative importance and relative feasibility considering all 73 implementation strategies. The benefit of concept mapping is the ability to quantitatively characterize how your target audience conceptualizes a wide range of topics. For the ERIC project, concept mapping provided us with a structured, participant driven approach to organizing our data into 9 expert-derived categories. This organization scheme was then used to structure additional expert panel tasks. This presentation will focus on concept mapping as a tool for characterizing an expert panel's shared understanding of key concepts to be used in a subsequent recommendation process. While data from the ERIC project will be used to illustrate this method, discussion will include how this method can be used to support active and structured stakeholder engagement in a variety of dissemination and implementation activities.

Development and application of a menu-based choice framework to structure expert recommendations for implementing complex practice changes in the VA

The Expert Recommendations for Implementing Change (ERIC) project sought to utilize methods to support a highly structured and transparent recommendation process that actively engaged key stakeholders throughout the project's execution. The ERIC project's penultimate activity involves a menu based choice (MBC) task. MBC methods are used in consumer marketing research for product development and these tasks are useful for providing a context rich structure for making decisions that involve multiple elements. In ERIC's MBC tasks, panelists were presented with 73 implementation strategies structured into nine categories. They were tasked with building multi-strategy implementation approaches for particular clinical practice changes being implemented across three settings, each with specific relative strengths and weaknesses (i.e., varying contextual characteristics).

The clinical practice changes were identified by national VA leadership as high priority areas for clinical quality improvement efforts (e.g., improving safety for patients taking antipsychotic medications, depression outcome monitoring in primary care mental health, prolonged exposure therapy for treating post-traumatic stress disorder). Scenarios describing these practice changes were developed using key informant interviews with front line providers, clinical managers, health service researchers, and implementation scientists. These experts all practice in the respective area and were able to provide common and realistic challenges they face in routine service delivery in VA settings. ERIC project staff then expanded the scenarios to address varying organizational contexts (e.g., organizational culture, leadership, evaluation infrastructure) and across levels of evidence (e.g., strength and quality, relative advantage, compatibility, adaptability). Stakeholders were repeatedly engaged in an iterative process of evaluating the scenarios for reliability, credibility, and transferability. This presentation will highlight the critical role partnering with key stakeholders plays in executing this structured recommendation method.

Primary funding for this research was provided by the U.S. Department of Veterans Affairs Veterans Health Administration's Mental Health Quality Enhancement Research Initiative (QLP 55-025).

